# Temporal trends, tumor characteristics and stage-specific survival in penile non-squamous cell carcinoma vs. squamous cell carcinoma

**DOI:** 10.1007/s10552-021-01493-3

**Published:** 2021-09-02

**Authors:** Mike Wenzel, Nicolas Siron, Claudia Collà Ruvolo, Luigi Nocera, Christoph Würnschimmel, Zhe Tian, Shahrokh F. Shariat, Fred Saad, Alberto Briganti, Derya Tilki, Severine Banek, Luis A. Kluth, Frederik C. Roos, Felix K. H. Chun, Pierre I. Karakiewicz

**Affiliations:** 1 Division of Urology, Cancer Prognostictables and Health Outcomes Unit, University of Montréal Health Center, Montreal, QC Canada; 2 Department of Urology, University Hospital Frankfurt, Goethe University Frankfurt, Theodor- Stern Kai 7, 60590 Frankfurt am Main, Germany; 3 Department of Neurosciences, Reproductive Sciences and Odontostomatology, University of Naples Federico II, Naples, Italy; 4 Department of Urology and Division of Experimental Oncology, URI, Urological Research Institute, IRCCS San Raffaele Scientific Institute, Milan, Italy; 5 Martini-Klinik Prostate Cancer Center, University Hospital Hamburg-Eppendorf, Hamburg, Germany; 6 Department of Urology, Comprehensive Cancer Center, Medical University of Vienna, Vienna, Austria; 7 Departments of Urology, Weill Cornell Medical College, New York, NY USA; 8 Department of Urology, University of Texas Southwestern, Dallas, TX USA; 9 Department of Urology, Second Faculty of Medicine, Charles University, Prag, Czech Republic; 10 Institute for Urology and Reproductive Health, I.M. Sechenov First Moscow State Medical University, Moscow, Russia; 11 Division of Urology, Department of Special Surgery, Jordan University Hospital, The University of Jordan, Amman, Jordan; 12 Department of Urology, University Hospital Hamburg-Eppendorf, Hamburg, Germany

**Keywords:** Penile cancer, Variant histology, Squamous cell carcinoma, CSM, Cancer-specific mortality, SCC, Adenocarcinoma, Melanoma

## Abstract

**Purpose:**

To compare Cancer-specific mortality (CSM) in patients with Squamous cell carcinoma (SCC) vs. non-SCC penile cancer, since survival outcomes may differ between histological subtypes.

**Methods:**

Within the Surveillance, Epidemiology and End Results database (2004–2016), penile cancer patients of all stages were identified. Temporal trend analyses, cumulative incidence and Kaplan–Meier plots, multivariable Cox regression and Fine and Gray competing-risks regression analyses tested for CSM differences between non-SCC vs. SCC penile cancer patients.

**Results:**

Of 4,120 eligible penile cancer patients, 123 (3%) harbored non-SCC vs. 4,027 (97%) SCC. Of all non-SCC patients, 51 (41%) harbored melanomas, 42 (34%) basal cell carcinomas, 10 (8%) adenocarcinomas, eight (6.5%) skin appendage malignancies, six (5%) epithelial cell neoplasms, two (1.5%) neuroendocrine tumors, two (1.5%) lymphomas, two (1.5%) sarcomas. Stage at presentation differed between non-SCC vs. SCC. In temporal trend analyses, non-SCC diagnoses neither decreased nor increased over time (*p* > 0.05). After stratification according to localized, locally advanced, and metastatic stage, no CSM differences were observed between non-SCC vs. SCC, with 5-year survival rates of 11 vs 11% (*p* = 0.9) for localized, 33 vs. 37% (*p* = 0.4) for locally advanced, and 1-year survival rates of 37 vs. 53% (*p* = 0.9) for metastatic penile cancer, respectively. After propensity score matching for patient and tumor characteristics and additional multivariable adjustment, no CSM differences between non-SCC vs. SCC were observed.

**Conclusion:**

Non-SCC penile cancer is rare. Although exceptions exist, on average, non-SCC penile cancer has comparable CSM as SCC penile cancer patients, after stratification for localized, locally invasive, and metastatic disease.

## Introduction

Penile cancer is a rare disease with an overall incidence rate of 0.8 per 100 000 persons in Europe and the United States [1–7]. Most diagnosed penile cancers (95%) are of Squamous cell carcinoma (SCC) histology. Other histologies (non-SCC) include basal cell carcinoma, melanoma, sarcoma or lymphoma [4, 8, 9].

Most contemporary large-scale epidemiological penile cancer studies exclusively focused on SCC histological subtype [4, 10–14]. Only one large-scale, and several case series and case reports examined outcomes of non-SCC penile cancer [15–20]. The largest single institutional cohort of non-SCC penile cancer (1996–2012) consisted of 12 patients with melanoma, sarcoma, and sebaceous carcinoma histologies [17]. In the large-scale analyses by Bhambhavi et al. (1975–2016, *n* = 666) relying on the Surveillance, Epidemiology and End Results (SEER) database, a large proportion of patients were diagnosed prior to year 2000 (42.2%). Of those with non-SCC, mostly harbored Kaposi sarcoma (27.5%, *n* = 183). Of these, only 43 patients were sampled in the SEER database after 2004 [20]. Finally, the study of Bhambhavi et al. did not exclude non-invasive non-SCC (Ta stage) or precursor lesions.

Therefore, their results might not reflect contemporary distribution of non-SCC penile cancer. In consequence, current trends and stage-specific survival analyses of non-SCC penile cancer are largely unknown. We addressed this void and relied on the SEER database (2004–2016). Since in urological malignancies rare histological subtypes are often associated with higher stage at diagnosis and higher Cancer-specific mortality (CSM), we hypothesized that non-SCC penile cancer may differ in stage-specific survival outcomes, relative to SCC histology [21–24]. This information may be important for clinicians in patient counseling and for therapy planning.

## Materials and methods

### Study population

The current SEER 18 database samples 35% of the United States population and approximates it in demographic composition and cancer incidence [25]. Within SEER database (2004 − 2016), we identified patients ≥ 18 years old with histologically confirmed primary penile cancer (International Classification of Disease for Oncology [ICD-O] site code C60.0). Histological subtype was defined as either SCC, basal cell, melanoma, skin appendage malignancy, neuroendocrine, adenocarcinoma, lymphoma, sarcoma or as epithelial neoplasm (including non-small cell carcinoma, pseudocarcinomatous carcinoma, and undifferentiated carcinoma), according to the WHO criteria [1, 9, 26]. Unknown histology, penile intraepithelial neoplasia and precursor lesions such as Paget’s disease were excluded. Cases identified only at autopsy or death certificate were also excluded. TNM-stage was used according the 7th AJCC edition [27]. According to SEER mortality code, CSM was defined as deaths related to penile cancer. All other deaths were considered as other cause mortality (OCM).

### Statistical analysis

Descriptive statistics included frequencies and proportions for categorical variables. Means, medians, and interquartile-ranges were reported for continuously coded variables. The Chi-square tested the statistical significance in proportions’ differences. The t-test examined the statistical significance of means’ and distributions’ differences.

To access temporal trends in non-SCC and SCC penile cancer, log linear regressions were used to compute estimated annual percent changes (EAPC), as previously described [28, 29]. Moreover, Kaplan–Meier plots were fitted to test the effects of non-SCC on CSM across different tumor stages of localized (T_1-2_N_0_M_0_), locally invasive (T_3-4_N_0_M_0_/T_1-2_N_1-3_M_0_), and metastatic stages (T_1-4_N_0-3_M_1_), relative to SCC penile cancer. Finally, univariable and multivariable Cox regression models were fitted to adjust for differences in patient and tumor characteristics.

Finally, propensity score matching was performed for age at diagnosis, tumor size, T-stage (T1 vs. T2 vs. T3 vs. T4), N-stage (N0/Nx vs. N1 vs. N2 vs. N3) and M-stage (M0/Mx vs. M1) with the objective to maximally reduce those differences with the intent of illustrating CSM, in a fashion that minimizes the contribution of other variables, except for non-SCC vs. SCC histology. Additionally, cumulative incidence plots addressed CSM after adjustment for OCM. In multivariable fine and gray Competing-risks regression (CRR) models covariates consisted of marital status, socioeconomic status, race/ethnicity, surgical treatment, lymph node dissection, chemotherapy, and radiation therapy.

All tests were two sided with a level of significance set at *p* < 0.05 and R software environment for statistical computing and graphics (version 3.4.3, Boston, United States) was used for all analyses.

## Results

### Descriptive characteristics of the study population

In 4,120 eligible penile cancer patients, 123 (3%) harbored non-SCC vs. 3997 (97%) SCC, respectively (Table 1). Overall median age at diagnosis was 68 years and overall median follow-up time was 30 months with no significant differences between non-SCC and SCC (both *p* > 0.6). In both non-SCC and SCC penile cancer, most patients were Caucasians (74.8 vs. 63.4%). Rates of Hispanics (11.4 vs. 20.6%) and African Americans (4.9 vs. 10.0%) were lower in non-SCC, relative to SCC penile cancer patients (*p* < 0.01).

**Table 1:** Descriptive characteristics of penile cancer patients

Variable	Overall *n* = 4,120	Non-SCC *n* = 123 (3%)	SCC *n* = 3,997 (97%)	*p*-value
Age, years				
Median (IQR)	68 (57–78)	69 (56–80)	68 (58–77)	0.6
Follow-up, months				
Median (IQR)	30 (11–70)	30(11–70)	38 (13–64)	0.7
Race/ethnicity				
African American	407 (9.9)	6 (4.9)	401 (10.0)	< 0.01
Caucasian	2,625 (63.7)	92 (74.8)	2,533 (63.4)	
Hispanic	839 (20.4)	14 (11.4)	825 (20.6)	
Other	249 (6.0)	11 (8.9)	238 (6.0)	
Region				
West	1,864 (45.2)	61 (49.6)	1,803 (45.1)	0.5
North-East	1,673 (40.6)	49 (39.8)	1,624 (40.6)	
Midwest	379 (9.2)	10 (8.1)	369 (9.2)	
Southwest	204 (5.0)	3 (2.4)	201 (5.0)	
T stage				
T1	2,319 (55.9)	62 (50.4)	2,257 (56.1)	< 0.001
T2	878 (21.2)	18 (14.6)	860 (21.4)	
T3	613 (14.8)	14 (11.4)	599 (14.9)	
T4	97 (2.3)	13 (10.6)	84 (2.1)	
Tx/Unknown	195 (4.7)	16 (13.0)	179 (4.4)	
N stage				
N0	3,088 (75.0)	91 (74.0)	2,997 (75.0)	< 0.001
N1	249 (6.0)	6 (4.9)	243 (6.1)	
N2	264 (6.4)	4 (3.3)	260 (6.5)	
N3	192 (4.7)	1 (0.8)	191 (4.8)	
NX/Unknown	311 (7.5)	21 (17.1)	290 (7.3)	
M stage				
M0	3,791 (92)	107 (87.0)	3,684 (92.2)	0.05
M1	142 (3.4)	6 (4.9)	136 (3.4)	
MX/Unknown	171 (4.2)	10 (8.1)	161 (4.0)	
Tumor size				
≤ 1 cm	254 (6.2)	15 (12.2)	239 (6.0)	< 0.001
1–5 cm	2,053 (49.8)	37 (30.1)	2,016 (50.4)	
> 5 cm	1,722 (41.8)	65 (52.8)	1,657 (41.5)	
Unknown	91 (2.2)	6 (4.9)	85 (2.1)	
Surgery				
No	376 (9.1)	13 (10.6)	363 (9.1)	0.2
Surgery/Local excision	3,723 (90.4)	108 (87.8)	3,615 (90.4)	
Unknown	21 (0.5)	2 (1.6)	19 (0.5)	
LND				
Yes	838 (20.3)	33 (26.8)	805 (20.1)	0.08
Radiation therapy				
Yes	353 (8.6)	3 (2.4)	350 (8.8)	0.03
Chemotherapy				
Yes	410 (10.0)	7 (5.7)	403 (10.1)	0.2

Descriptive characteristics of 4,120 penile cancer patients stratified according to non-squamous cell carcinoma (Non-SCC) vs squamous cell carcinoma (SCC) histology identified within the Surveillance, Epidemiology, and End Results database (2004–2016)

*IQR* interquartile range, *LND* Lymph node dissection

No regional variations in prevalence between non-SCC and SCC were observed (*p* = 0.5). According to stage at presentation, most patients were diagnosed at a localized stage. Specifically, when comparing non-SCC to SCC patients, 65 vs. 78% harbored T1–2 stage (*p* < 0.001). Conversely, non-SCC patients more often harbored T3–4 stage (22.0 vs. 17.0%) at presentation (*p* < 0.001). No significant differences were observed in metastatic stage at presentation between non-SCC vs. SCC (4.9 vs. 3.4%, *p* = 0.05). Patients with SCC more frequently underwent radiation therapy than non-SCC patients (8.8 vs. 2.4%, *p* = 0.03).

Of 123 non-SCC penile cancer patients (Table 2), the most prevalent histological subtypes were melanoma (*n* = 51, 41%) and basal cell carcinoma (*n* = 42, 34%). The remaining non-SCC consisted of either adenocarcinoma (*n* = 10, 8%), skin appendage malignancies (*n* = 8, 6.5%), epithelial neoplasm (*n* = 6, 5%), neuroendocrine tumors (*n* = 2, 1.5%), lymphomas (*n* = 2, 1.5%) or sarcomas (*n* = 2, 1.5%, Table 2). Median age ranged from 66 (adenocarcinoma) to 83 (neuroendocrine tumor). Proportions of Hispanics (40%, *n* = 4) and African Americans (30%, *n* = 3) were highest in adenocarcinoma non-SCC penile cancer subtype.

**Table 2:** Descriptive characteristics of penile cancer patients according to variant histologies

Variable								Epithelial histology		
	Melanoma *n* = 51 (41%)	Basal cell *n* = 42 (34%)	Adeno-carcinoma *n*= 10 (8%)	Skin appendage malignancy *n* = 8 (6.5%)	Neuro-endocrine *n* = 2 (1.5%)	Lymphoma *n* = 2 (1.5%)	Sarcoma *n* = 2 (1.5%)	Non-small cell carcinoma *n* = 1	Pseudo-sarcomatous carcinoma *n* = 4	Undifferentiated, carcinoma *n* = 1
Age, years										
Median (IQR)	69 (58–80)	69 (55–80)	66 (56–73)	64 (49–78)	83 (82–85)	67 (56–78)	69 (68–69	87 (NA)	70 (67–70)	79 (NA)
Follow-up, months										
Median (IQR)	37 (17–63)	45 (18–63)	27 (11–60.5)	75 (42–99)	5 (4–5)	52 (50–53)	8.5 (7–10)	2 (NA)	8.5 (6–15)	65 (NA)
Race/ethnicity										
African-American	1 (2.0)	1 (2.4)	3 (30)	0 (0)	0 (0)	0 (0)	0 (0)	0 (0)	0 (0)	1 (100)
Caucasian	38 (74.5)	37 (88.1)	3 (30)	6 (75)	2 (100)	2 (100)	1 (50)	1 (100)	2 (50)	0 (0)
Hispanic	6 (11.8)	2 (4.8)	4 (40)	1 (12.5)	0 (0)	0 (0)	0 (0)	0 (0)	1 (25)	0 (0)
Other	6 (11.8)	2 (4.8)	0 (0)	1 (12.5)	0 (0)	0 (0)	1 (50)	0 (0)	1 (25)	0 (0)
Region										
West	28 (54.9)	19 (45.2)	4 (40)	4 (50)	1 (50)	1 (50)	1 (50)	1 (100)	2 (50)	0 (0)
North-East	20 (39.2)	18 (42.9)	4 (40)	3 (37.5)	0 (0)	1 (50)	1 (50)	0 (0)	2 (50)	0 (0)
Midwest	2 (3.9)	4 (9.5)	1 (10)	1 (12.5)	1 (50)	0 (0)	0 (0)	0 (0)	0 (0)	1 (100)
Southwest	1 (2)	1 (2.4)	1 (10)	0 (0)	0 (0)	0 (0)	0 (0)	0 (0)	0 (0)	0 (0)
T stage										
T1	12 (23.5)	30 (71.4)	6 (60)	7 (87.5)	1 (50)	2 (100)	0 (0)	1 (100)	2 (50)	1 (100)
T2	12 (23.5)	2 (4.8)	3 (30)	0 (0)	1 (50)	0 (0)	0 (0)	0 (0)	0 (0)	0 (0)
T3	13 (25.5)	0 (0)	0 (0)	0 (0)	0 (0)	0 (0)	0 (0)	0 (0)	1 (25)	0 (0)
T4	11 (21.6)	2 (4.8)	0 (0)	0 (0)	0 (0)	0 (0)	0 (0)	0 (0)	0 (0)	0 (0)
Tx/Unknown	3 (5.9)	8 (19)	1 (10)	1 (12.5)	0 (0)	0 (0)	2 (100)	0 (0)	1 (25)	0 (0)
N stage										
N0	40 (78.4)	27 (64.3)	10 (100)	7 (87.5)	1 (50)	1 (50)	0 (0)	1 (100)	3 (75)	1 (100)
N1	5 (9.8)	0 (0)	0 (0)	0 (0)	0 (0)	0 (0)	0 (0)	0 (0)	1 (25)	0 (0)
N2	3 (5.9)	1 (2.4)	0 (0)	0 (0)	0 (0)	0 (0)	0 (0)	0 (0)	0 (0)	0 (0)
N3	0 (0)	0 (0)	0 (0)	0 (0)	1 (50)	0 (0)	0 (0)	0 (0)	0 (0)	0 (0)
NX/Unknown	3 (5.9)	14 (33.3)	0 (0)	1 (12.5)	0 (0)	1 (50)	2 (100)	0 (0)	0 (0)	0 (0)
M stage										
M0	47 (92.2)	35 (83.3)	9 (90)	7 (87.5)	2 (100)	1 (50)	0 (0)	1 (100)	4 (100)	1 (100)
M1	3 (5.9)	2 (4.8)	1 (10)	0 (0)	0 (0)	0 (0)	0 (0)	0 (0)	0 (0)	0 (0)
MX/Unknown	1 (2)	5 (11.9)	0 (0)	1 (12.5)	0 (0)	1 (50)	2 (100)	0 (0)	0 (0)	0 (0)
Tumor size										
≤ 1 cm	7 (13.7)	6 (14.3)	1 (10)	1 (12.5)	0 (0)	0 (0)	0 (0)	0 (0)	0 (0)	0 (0)
1–5 cm	17 (33.3)	10 (23.8)	4 (40)	2 (25)	1 (50)	0 (0)	2 (100)	0 (0)	0 (0)	1 (100)
> 5 cm	25 (49)	23 (54.8)	5 (50)	5 (62.5)	1 (50)	2 (100)	0 (0)	1 (100)	3 (75)	0 (0)
Unknown	2 (3.9)	3 (7.1)	0 (0)	0 (0)	0 (0)	0 (0)	0 (0)	0 (0)	1 (25)	0 (0)
Surgery										
Surgery/Local excision	49 (96.1)	34 (81)	7 (70)	7 (87.5)	2 (100)	2 (100)	2 (100)	1 (100)	3 (75)	1 (100)
No	2 (3.9)	6 (14.3)	3 (30)	1 (12.5)	0 (0)	0 (0)	0 (0)	0 (0)	1 (25)	0 (0)
Unknown	0 (0)	2 (4.8)	0 (0)	0 (0)	0 (0)	0 (0)	0 (0)	0 (0)	0 (0)	0 (0)
LND										
Yes	28 (54.9)	1 (2.4)	1 (10)	0 (0)	1 (50)	0 (0)	0 (0)	0 (0)	2 (50)	0 (0)
Radiation therapy										
Yes	0 (0)	1 (2.4)	2 (20)	0 (0)	0 (0)	0 (0)	0 (0)	0 (0)	0 (0)	0 (0)
Chemotherapy										
Yes	2 (3.9)	1 (2.4)	2 (20)	0 (0)	1 (50)	0 (0)	0 (0)	0 (0)	1 (25)	0 (0)

Descriptive characteristics of 123 non-squamous cell carcinoma penile cancer patients, namely, melanoma, basal cell carcinoma, skin appendage malignancy, neuroendocrine, adenocarcinoma, lymphoma, sarcoma, and epithelial neoplasms (including non-small cell carcinoma, pseudosarcomatous carcinoma, and undifferentiated carcinoma)

*IQR* interquartile range, *LND* lymph node dissection, *NA* not available

### Temporal trend analysis of non-SCC vs. SCC penile cancer

**Fig. 1:**
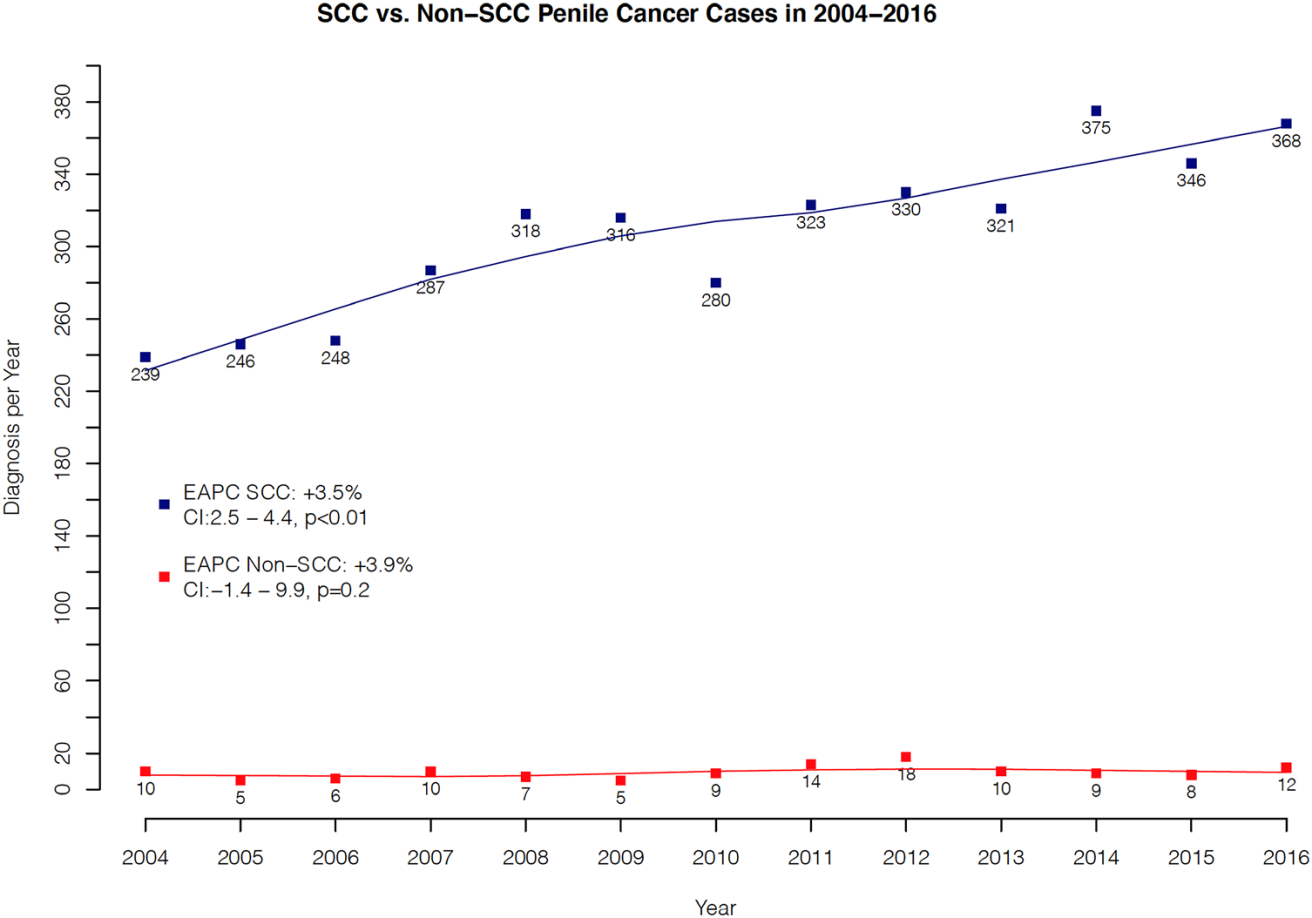
Estimated annual percent change (EAPC) of rates in Squamous cell (SCC) and Non-squamous cell penile cancer (Non-SCC). *CI* confidence interval

In temporal trends, non-SCC penile cancer did not change over time (EAPC + 3.9%, CI − 1.4 to 9.9, *p* = 0.2), with a range of five to 18 cases per year (Fig. 1). Conversely, in trend analyses regarding SCC penile cancer, increasing rates over time from 2004 to 2016 with an EAPC of + 3.5% (CI 2.5–4.4, *p* < 0.01) were recorded and cases ranged from 239 to 368 per year.

### CSM in non-SCC penile cancer

Differences in CSM between non-SCC vs. SCC penile cancer exist. Specifically, unadjusted five-year CSM rates were 0% for skin appendage malignancies (*n* = 8) vs. 8.8% for basal cell carcinoma (*n* = 42) vs. 12.5% for adenocarcinoma (*n* = 10) vs. 20% for epithelial neoplasms (*n* = 6) and 20.5% for SCC (*n* = 3,997). Finally, melanoma (*n* = 51) patients exhibited the highest 5-year CSM rate with 31.2%. CSM could not be computed for lymphoma and sarcoma penile cancer patients (both *n* = 2), since incomplete follow-up was available. Of two neuroendocrine patients (*n* = 2), one patient died at 4 months, while the second survived until five months and was lost to follow-up.

### CSM in localized, locally advanced and metastatic non-SCC

**Fig. 2:**
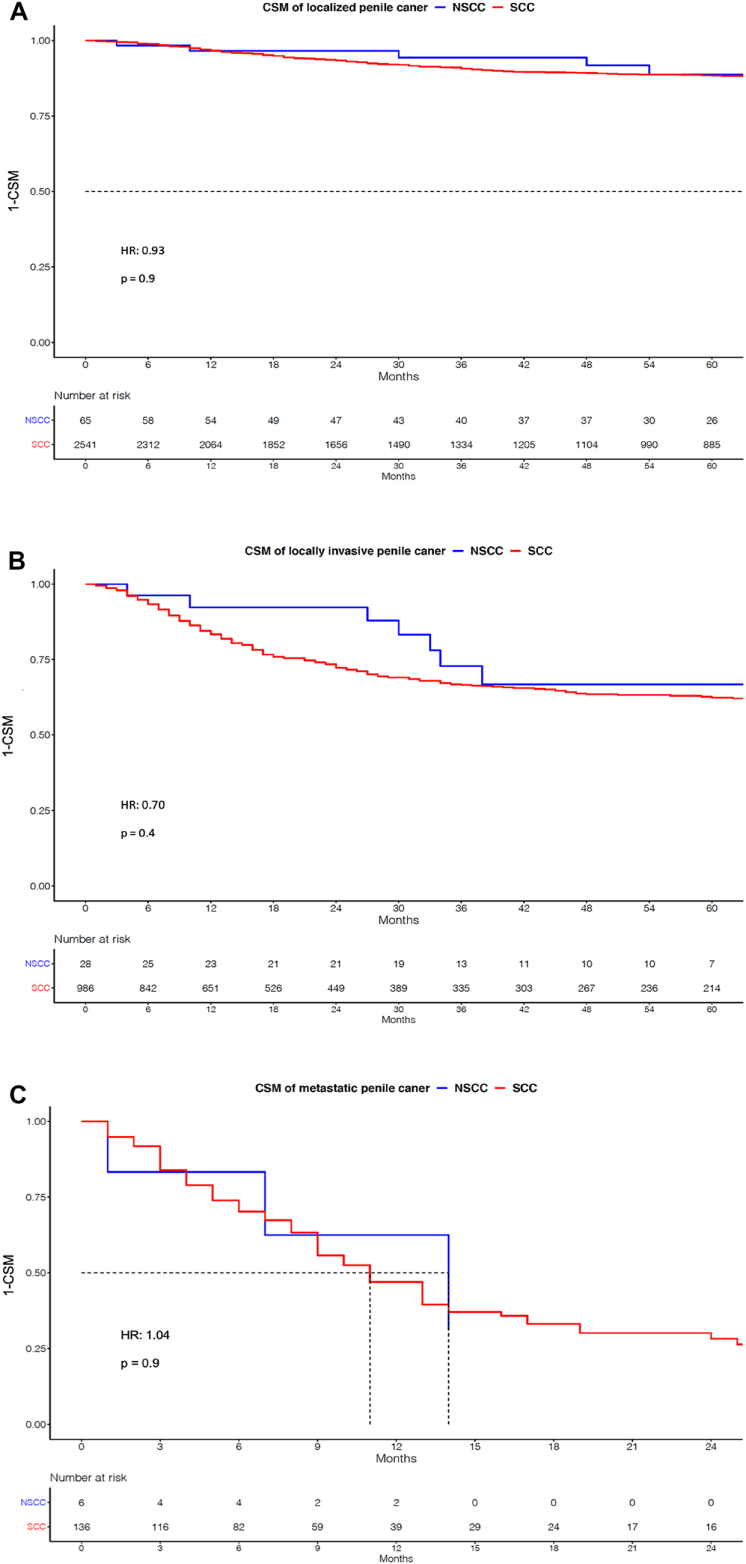
Kaplan–Meier plots depicting Cancer specific mortality (CSM) in Non-squamous cell carcinoma (NSCC) vs. Squamous cell carcinoma (SCC) in **A** localized penile cancer, **B** locally invasive penile cancer, and **C** metastatic penile cancer. *HR* hazard ratio

We performed additional separate comparisons between all non-SCC vs. SCC histologies for (A) localized, (B) locally advanced, and (C) metastatic penile cancer stages, since stage at diagnosis significantly differed between both groups (Table 1; Fig. 2). In localized penile cancer, five-year CSM rates were 11% for both non-SCC (*n* = 65) vs. SCC (*n* = 2,541; hazard ratio [HR] 0.93, *p* = 0.9). Similarly, in locally advanced penile cancer, non-SCC (*n* = 28) vs. SCC (*n* = 986) five-year CSM rates were 33 vs. 37% (HR 0.7, *p* = 0.4). Finally, in metastatic penile cancer one-year CSM rates were 37 vs. 53% for non-SCC (*n* = 6) vs. SCC penile cancer (*n* = 136; HR 1.04, *p* = 0.9) respectively. Even after multivariable adjustment for age, T stage, and surgical treatment, no CSM differences were identified in all three stage-specific comparisons between non-SCC vs. SCC (all *p* > 0.05).

### The effect of non-SCC on CSM after propensity score matching

**Fig. 3:**
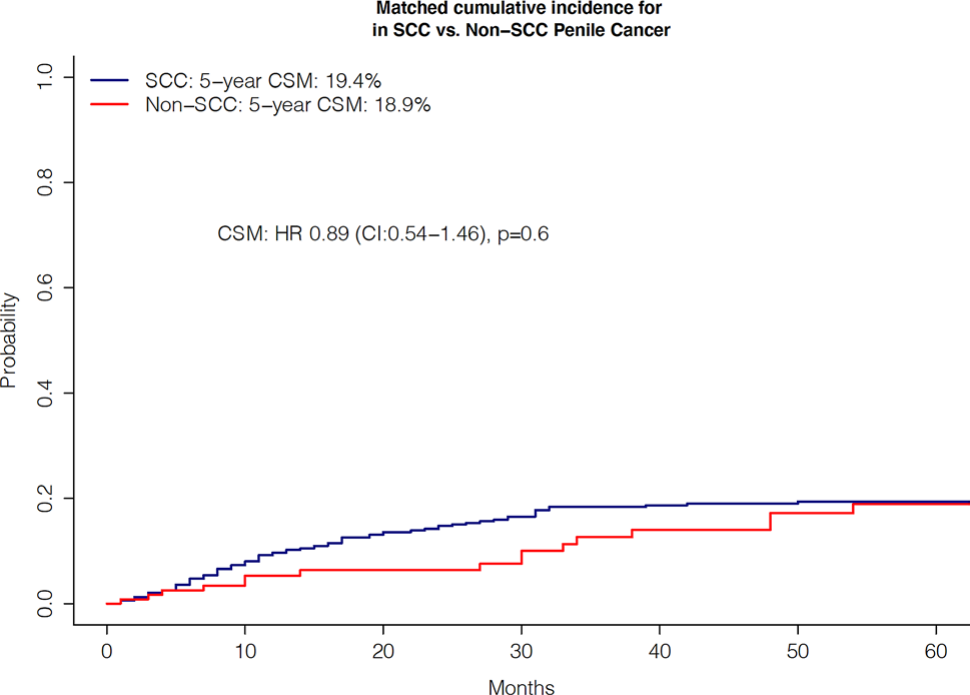
Cancer specific mortality (CSM) of Squamous cell (SCC) vs Non-squamous cell carcinoma (Non-SCC) penile cancer. Cumulative incidence plots depicting CSM after 4:1 propensity matching for age, tumor size, TNM stage, and surgery for SCC vs Non-SCC penile cancer. *HR* hazard ratio, *CI* confidence interval

Additionally, we further validated CSM differences between non-SCC vs. SCC penile cancer in cumulative incidence plots after 1:4 propensity score matching for age, tumor size, TNM stage, and surgery. The matched cumulative incidence CSM rates at five-years were 18.9 vs. 19.4% for non-SCC vs. SCC penile cancer, resulting in a HR of 0.74 (*p* = 0.4, Fig. 3). To maximally reduce the contribution of other variables, except for non-SCC vs. SCC histology, further matched multivariable competing risk regression analyses accounted for OCM and also adjusted for race/ethnicity, surgery, lymph node dissection, chemotherapy, and radiation therapy. Here, no CSM difference between non-SCC and SCC was observed (HR 1.1, *p* = 0.8, Table 3).

**Table 3:** Univariable and multivariable competing-risks regression models for penile cancer patients (squamous cell carcinoma [SCC] vs non-squamous cell carcinoma [non-SCC])

Variable	Univariable		Multivariable	
	HR (95% CI)	*p* value	HR (95% CI)	*p* value
Histology				
SCC	1.00 (Ref.)	–	1.00 (Ref.)	–
Non-SCC	0.89 (0.54–1.46)	0.6	1.1 (0.62–1.84)	0.8
Marital status				
Married	1.00 (Ref.)	–	1.00 (Ref.)	–
Unmarried	1.58 (1.05–2.37)	0.029	1.4 (0.92–2.14)	0.1
Unknown	0.54 (0.24–1.21)	0.1	0.69 (0.3–1.57)	0.4
Socioeconomic status				
1st quartile	1.00 (Ref.)	–	1.00 (Ref.)	–
2-4th quartile	1.79 (1.03–3.13)	0.04	1.64 (0.91–2.94)	0.1
Race/ethnicity				
Caucasian	1.00 (Ref.)	–	1.00 (Ref.)	–
African-American	1.43 (0.77–2.64)	0.3	0.91 (0.49–1.7)	0.7
Hispanic	1.18 (0.73–1.92)	0.5	0.95 (0.55–1.62)	0.8
Other	0.4 (0.13–1.27)	0.1	0.39 (0.13–1.15)	0.08
LND				
No	1.00 (Ref.)	–	1.00 (Ref.)	–
Yes	2.1 (1.4–3.15)	< 0.005	1.77 (1.13–2.78)	0.01
Chemotherapy				
No	1.00 (Ref.)	–	1.00 (Ref.)	–
Yes	4.21 (2.63–6.73)	< 0.005	2.6 (1.44–4.68)	< 0.005
Radiation				
No	1.00 (Ref.)	–	1.00 (Ref.)	–
Yes	4.33 (2.65–7.06)	< 0.005	2.61 (1.45–4.72)	< 0.005
Unknown	0 (0–0)	0	0 (0–0)	0

Univariable and multivariable competing-risks regression models for penile cancer patients after matching for age, tumor size, TNM stage, and surgery performance

*HR* hazard ratio, *CI* confidence interval, *LND* lymph node dissection

## Discussion

We hypothesized that contemporary non-SCC penile cancer patients may differ from historical cases that were heavily weighted towards Kaposi’s sarcomas. Moreover, we tested for CSM differences between non-SCC and SCC penile cancer patients and made several important observations.

First, we recorded important baseline characteristic differences in non-SCC vs. SCC penile cancer patients. Specifically, we noted a median age at diagnosis of 68 years, highest prevalence in Caucasians (75%), and most diagnoses occurring at a localized stage (65%). These observations are particularly different from the population-based report by Bhambhvani et al., relying on the SEER database 1975–2016, in which over 42% of patients were diagnosed prior to year 2000 and without excluding non-invasive and precursor lesions [20]. For example, in this more historical report, mean age of non-SCC patients was 61 years. Moreover, proportions of Caucasians were higher and rates of localized disease in non-SCC were lower. However, relative to SCC, rates of localized SCC were higher than in non-SCC penile cancer patients (77.5 vs. 65%) in the current study. Despite an expected result, this finding is noteworthy to consider for clinicians when patients with non-SCC penile cancer are counseled and treatment decision making is done.

Second, we made important observations regarding non-SCC penile cancer histologies. The most frequent non-SCC histology were melanomas (41%), followed by basal cell carcinomas (34%). This observation differed from historical observations that were heavily weighted towards Kaposi’s sarcomas. It is also of note that within non-SCC cohort, patient characteristics strongly differed between histological subtypes. Specifically, age of adenocarcinoma patients was 66 years vs. 83 years of neuroendocrine patients. Moreover, respectively 40 and 30% of adenocarcinoma non-SCC patients were Hispanic and African Americans. Conversely, all neuroendocrine non-SCC patients were Caucasian. To the best of our knowledge, we are the first to report on non-SCC characteristics within individual histological subtypes. In consequence, our data cannot be directly compared to previous publications. However, in the population-based report by Bhambhvani et al. most non-SCC patients harbored Kaposi’s sarcomas, followed by melanomas and basal cell carcinomas [20]. Within the current analyses, we exclusively focused on most contemporary patients (2004–2016) with non-SCC and excluded non-invasive and precursor lesions. Interestingly, no Kaposi’s sarcoma patients were identified. This observation is important and validates the SEER database in the context of its accuracy even in very rare diagnosis.

Third, we also made important observations regarding CSM stage-specific comparisons between non-SCC vs. SCC patients. Specially, we identified no significant differences in CSM between non-SCC vs. SCC penile cancers across all penile cancer stages. This was further validated in propensity score matched and multivariate competing risks regression analyses. However, non-SCC penile cancers are a heterogeneous group of variant histologies. In consequence, it is possible that specific non-SCC penile cancer histologies may exhibit more or less favorable outcomes relative to SCC penile cancer patients. For example, penile melanoma and genitourinary sarcoma are known to be very aggressive cancers and exhibit poor prognosis and have high disease recurrence, even after surgery [16–19, 30–32]. In our study, we reported a five-year CSM of 31.2% in penile melanoma (*n* = 51). However, due to small sample size, comparisons between individual variant non-SCC penile cancers histologies, such as penile melanoma and sarcoma (*n* = 2), could not be directly compared to SCC. In the population-based report by Bhambhvani et al., melanoma non-SCC patients diagnosed between 1975 and 2016 displayed a CSM disadvantage relative to SCC patients. The disadvantage persisted even in the most recent subgroup of patients diagnosed between 2000 and 2016 [20]. Conversely, relying on the same methodology, no CSM differences were recorded between basal cell carcinoma vs. SCC penile cancer patients diagnosed between 2000 and 2016. These observations indicate specific patterns within different variant histologies in non-SCC patients. However, our data suggest that on average and as a whole, non-SCC patients display similar CSM as SCC patients.

Fourth, we observed that the temporal trends of non-SCC penile cancer did not change over time between 2004 and 2016. Conversely, rates of SCC penile cancer increased with an EAPC of + 3.5% per year between 2004 and 2016. This trend is contrary to the more historically reported negative trend in overall age-adjusted SCC incidence rates between 1973 and 2002 in the United States, where Barnholtz-Sloan et al. relied on the SEER database [4]. In comparison, there has been increased incidence in Denmark, the United Kingdom, Germany, and Norway [6, 7, 33, 34]. For example, in Germany, Schoffer et al. noted an age-standardized incidence rate of penile cancer of 1.2 per 100,000 in 1961 compared to 1.8 per 100,000 in 2012, with a corresponding increase in EAPC of + 4.6% (CI 0.62–8.86) between 2003 and 2012. Similarly, in Norway, Hansen noted an increased EAPC of + 0.8% (CI 0.46–1.15) between 1956 and 2015. Given that penile cancer incidence increases with age, this tendency may partly be explained by increasing life-expectancy of the populations in these countries [1, 2].

Taken together, our study demonstrated that non-SCC and SCC penile cancer patients have similar population characteristics in terms of age at diagnosis or regions. However, differences were observed in stage at presentation. Furthermore, we observed no differences in five-year CSM between non-SCC vs. SCC penile cancer across localized, locally advanced, and metastatic stages. However, non-SCC penile cancer represents a heterogenous group of variant histologies. In consequence, individual histologies may have relatively more or less favorable CSM compared to SCC. Finally, we observed no increase in non-SCC penile cancer rates over time, but an increase in temporal trends of SCC penile cancer.

Our work has limitations and sample sizes of variant histologies of penile cancer were small and made it impossible to perform matched and multivariate CSM comparisons according to individual variant histologies. Moreover, limitations in sample sizes may precluded some analyses to provide statistically significant or meaningful p-values. Because of these small sample sizes, we grouped patients within variant histologies. As a result, it is possible that some of these individual histologies may have more favorable survival than others or vice versa. In consequence, specific conclusions regarding comparisons of CSM between individual histologies cannot be made. Finally, we were unable to adjust for tumor grade, despite it being recognized as a significant prognostic factor, as there is no established standardized grading for variant histologies [1].

## Conclusion

Non-SCC penile cancer is rare. Although exceptions exist, on average, non-SCC penile cancer has comparable CSM as SCC penile cancer patients, after stratification for localized, locally invasive, and metastatic disease.

## Data Availability

All datasets will be made available upon request for bona fide researchers.

## Code availability

All datasets will be made available upon request for bona fide researchers. Software R statistics (version 3.6.1).
